# Effects of Climatic Conditions and Supplementation with Palm Cake on the Thermoregulation of Crossbred Buffaloes Raised in a Rotational Grazing System and with Natural Shade in Humid Tropical Regions

**DOI:** 10.3390/ani14010053

**Published:** 2023-12-22

**Authors:** Carolina Carvalho Brcko, Jamile Andrea Rodrigues da Silva, Alexandre Rossetto Garcia, André Guimarães Maciel e Silva, Lucieta Guerreiro Martorano, Reíssa Alves Vilela, Benjamim de Souza Nahúm, Antônio Vinícius Corrêa Barbosa, Welligton Conceição da Silva, Thomaz Cyro Guimarães de Carvalho Rodrigues, Éder Bruno Rebelo da Silva, José de Brito Lourenço-Júnior

**Affiliations:** 1 Postgraduate Program in Animal Science (PPGCAN), Institute of Veterinary Medicine, Federal University of Pará (UFPA), Federal Rural University of the Amazônia (UFRA), Brazilian Agricultural Research Corporation (EMBRAPA), Castanhal 68746-360, Brazil; carolbrcko@gmail.com (C.C.B.); andregms@gmail.com (A.G.M.e.S.); reissa@usp.br (R.A.V.); thomazguimaraes@yahoo.com.br (T.C.G.d.C.R.); eder.b.rebelo@gmail.com (É.B.R.d.S.); joselourencojr@yahoo.com.br (J.d.B.L.-J.); 2 Institute of Animal Health and Production, Federal Rural University of the Amazônia (UFRA), Belem 66077-830, Brazil; jamileandrea@yahoo.com.br (J.A.R.d.S.); profvinibarbo@gmail.com (A.V.C.B.); 3 Brazilian Agricultural Research Corporation, Embrapa Southeast Livestock, Rod Washington Luiz, São Carlos 13560-970, Brazil; alexandre.garcia@embrapa.br; 4 Embrapa Eastern Amazon, Belem 66095-903, Brazil; lucieta.martorano@embrapa.br (L.G.M.); benjamim.nahum@embrapa.br (B.d.S.N.)

**Keywords:** animal bioclimatology, equatorial climate, co-product, heat stress, supplement, ruminants, thermoregulation

## Abstract

**Simple Summary:**

The objective of this work was to evaluate the effects of climatic conditions and supplementation based on palm kernel cake, on the thermoregulation of crossbred buffaloes in the eastern Amazon. Over a 12-month period, 24 female buffaloes with an initial age of 54 ± 7 months and an average weight of 503.1 ± 23 kg were divided into four groups with different levels of palm kernel cake supplementation relative to body weight. The animals were kept in *Brachiaria brizantha* pastures with access to water and mineral salt. Supplementation did not influence the physiological variables of thermoregulation. Throughout the year, mean values of rectal temperature, respiratory rate and body surface temperature were higher in the afternoon (*p* > 0.05). Supplementation with palm cake did not result in influences on the thermoregulation of buffaloes in the study region. The respiratory rate showed an association with the annual seasonality of temperatures, with higher averages in the afternoons of the rainy season. The positive correlation between the rectal temperature, respiratory rate, and body surface temperature indicates that buffaloes respond to atmospheric thermal elevations (afternoon period), which is reflected in increasing thermal indices.

**Abstract:**

In ruminants, diet composition has a positive correlation with heat production, which can influence thermoregulation, energy expenditure and, consequently, animal performance. The objective of this work was to evaluate the effects of climatic conditions and supplementation based on palm kernel cake, on the thermoregulation of crossbred buffaloes in the eastern Amazon. The research was carried out at Embrapa Amazônia Oriental (01°26′ S and 48°24′ W), Belém, Pará, and lasted 12 months (representing the entire year). Twenty-four buffaloes, females, with initial age and an average weight of 54 ± 7 months and 503.1 ± 23 kg, respectively, non-pregnant, non-lactating and clinically healthy were used, divided into four treatments based on the supplementation content of the palm cake (%DM) in relation to their body weight (%): 0, 0.25, 0.50 and 1.0. The animals were kept in paddocks with *Brachiaria brizantha* (cv. Marandu), in a rotating system, with water to drink and mineral salt ad libitum. Equipment was installed to record environmental data (temperature and relative humidity, dew point temperature, wet bulb and black globe) and physiological data: rectal temperature (RT); respiratory rate (RR); and body surface temperature (BST), recorded twice a day, always in the morning (6:00 a.m. to 7:00 a.m.) and afternoon (12:00 p.m. to 1:00 p.m.) shifts, and were used to calculate the Globe Temperature and Humidity Index (GTHI). Supplementation did not influence the physiological variables of thermoregulation (*p* > 0.05). However, there were differences in the GTHI between the shifts, with higher means in the afternoon shift, especially in the less rainy period of the year, where the GTHI reached 92.06 ± 2.74 (*p* < 0.05). In all periods of the year, the mean values of RT, RR and BST were higher in the afternoon shift (*p* > 0.05). The respiratory rate (RR) is associated with the annual seasonality of the thermal waters, with higher averages in the afternoons of the rainy season. The positive correlation for rectal temperature, respiratory rate and body surface temperature indicated that buffaloes respond to thermal elevations in the atmosphere (afternoon period) and, consequently, reflect on the GTHI. Supplementation does not influence thermoregulation; the changes observed occurred in response to the region’s thermal and rainfall conditions (mainly in the afternoon shift), with higher GTHI values.

## 1. Introduction

Animal thermoregulation is a set of mechanisms that seek to adjust the internal body temperature of animals, in an attempt to maintain it at values compatible with life, when there is fluctuation in the temperature of the environment [1,2]. The use of these mechanisms has a direct influence on the animal’s behavior, energy expenditure and performance [3]. Some factors, such as diet, in a challenging climate situation, can also influence or enhance the thermoregulation process, due to the caloric increment [4]. Thermal stress can be caused by numerous factors such as skin lesions, no trees, and intense solar radiation [5,6].

Thermal comfort is defined as a state in which thermal equilibrium is zero. According to specifications of specific interest, the thermal comfort zone can be defined as between 16 and 25 °C, that is, in this ambient temperature range basal metabolism is lower and thus thermoregulation occurs without evapotranspiration [7].

This fact has already been verified in works with cattle [8,9], goats [10], sheep [11,12] and other species [13,14]. In the Amazon region, where the climate is predominantly equatorial, the largest buffalo herd in Brazil is located [15]. In this area, in addition to the high temperature and humidity, there is a period of more intense rainfall and a dry period during the year [16,17], where animals need to be supplemented to maintain body performance [18,19].

As an ingredient in supplements, palm kernel cake has been widely studied [20,21,22], as it is a low-cost co-product when compared to traditional ingredients, and has good nutritional value (especially protein and energy); the state of Pará is the largest producer of this material [23,24]. However, the works do not consider buffaloes and thermoregulatory responses, especially in the Amazon region, where most of the animals raised in the country are concentrated and which experience challenging climatic conditions.

Food plays a fundamental role in the thermoregulation of buffaloes, as it is directly related to the ability of these animals to regulate their body temperature. Buffaloes, especially in hot climates, face significant challenges, dissipating excessive heat. The digestion and metabolism of food, along with diet composition, affect internal heat production, which plays a vital role in regulating body temperature in buffaloes.

In buffaloes, thermal stress occurs due to specific factors that provide major disadvantages in relation to cattle, such as a black skin color, black hair, a reduced number of sweat glands/skin area, thick layer of skin epidermis, which makes it vulnerable to solar radiation [25,26].

In view of this, despite the need for animal supplementation in the dry period of the eastern Amazon, as nutritional changes have a direct influence on the thermoregulatory responses of ruminants, the inclusion of palm cake as a food supplement should be evaluated. Our hypothesis is that climatic conditions and the inclusion of a palm cake-based supplement in the diet may contribute to changes in the thermoregulation mechanisms of Buffaloes. Therefore, the objective of this work was to evaluate the effects of climatic conditions and palm cake-based supplementation of palm oil, on the thermoregulation of crossbred buffaloes in the eastern Amazon.

## 2. Materials and Methods

### 2.1. Ethical Aspects

The research was approved by the Ethics Committee for the Use of Animals (CEUA) of the Federal University of Pará—protocol BIO 120-13.

### 2.2. Experimental Area

**Figure 1:**
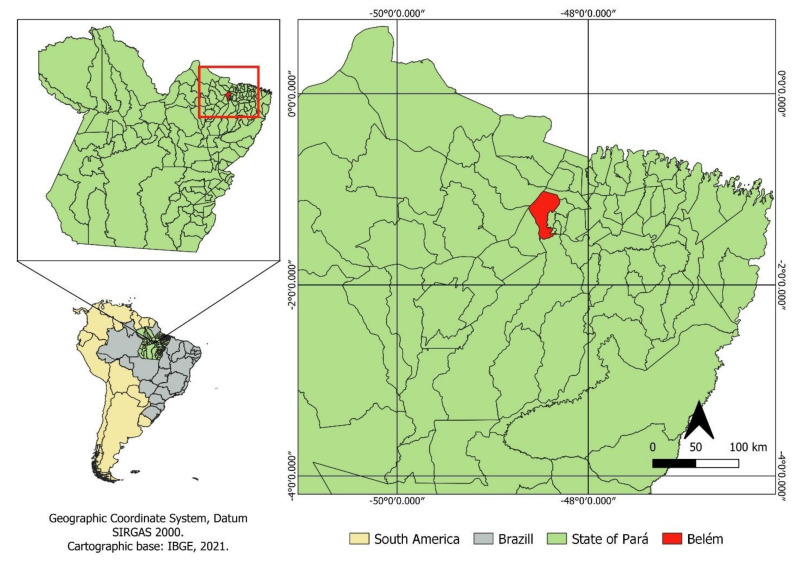
Location of the study area.

The experimental test was carried out at Embrapa Amazônia Oriental (01°26′ S and 48°24′ W) in Belem, Pará, (Figure 1), in the period from July 2013 to June 2014, during the rainy season of the year (January to April), specifically from February to March. The climate is tropical rainy Am, according to the Köppen classification, with an average annual rainfall of 2500 mm, an average temperature of 27 °C, and a relative humidity of 85%, and it has an Af2 climate, according to the Köppen classification adapted by Martorano et al. [27].

The animals were kept in paddocks with *Brachiaria Brizantha* grass (CV. Marandu) in a rotational system, natural shading, access to water and mineral salt ad libitum. They were distributed in a completely randomized design into four treatments, based on the level of inclusion of palm kernel cake in relation to their body weight: 0.0%, 0.25%, 0.50% and 1%. A total of 0.15% wheat bran was added to all treatments, acting as a palatability agent, and the supplement was offered in individual troughs, once a day, at 7:00 a.m. The forage supply was homogeneous in all paddocks, being around 9 kg of DM/100 kg of animal live weight throughout the experimental period.

### 2.3. Animals, Diets and Husbandry Conditions

Twenty-four crossbred buffaloes (Murrah × Mediterranean—*Bubalus bubalis bubalis*), with an initial age and average weight of 54 ± 7 months and 503.1 ± 23 kg, respectively, which were non-pregnant, non-lactating and clinically healthy were used.

Chemical analyses of the experimental diets were carried out at the Animal Nutrition Laboratory of the Federal University of Pará—UFPA. The content of dry and organic matter, ash and crude protein (Kjeldahl method) was in accordance with the recommendations of the AOAC [28]. Neutral detergent fiber (NDF), acid detergent fiber (ADF), cellulose and lignin followed the sequential method, described by Van Soest et al. [29]. The results are presented in Table 1.

### 2.4. Data Collect

An analysis of the thermal–hydric regime was carried out, using data from climatological normals, as well as meteorological data provided by INMET, corresponding to the experimental period.

Agrometeorological data were obtained from a portable automatic station, programmed to acquire data and store sensor readings in dataloggers every 1 min (Model TGD-300, Instrutherm, São Paulo, Brazil). The station was equipped with sensors for routine measurements of time and climate and a black globe thermometer, installed in each paddock, at the height of the back of the animals, in order to monitor the rotation of the animals in the pastures. The readings of the air temperature (AT, °C), relative air humidity (RH, %), dew point temperature (DPT, °C), wet bulb temperature (WBT, °C) and black globe temperature (BGT, °C) were performed from 6:00 a.m. to 7:00 a.m. and from 12:00 p.m. to 1:00 p.m., when the physiological variables were collected.

From the values of environmental variables, the Globe Temperature and Humidity Index (GTHI) was calculated, proposed by Buffington et al. [30], through the equation GTHI = BGT + 0.36 TPO + 41.5. The GTHI value considers the effects of dry bulb temperature, RH, solar radiation and air movement, since the BGT, which composes the formula of this index, indicates the effects of the combination of these meteorological variables [31], in addition to providing an indirect measure of ambient radiant heat. GTHI values are used to assess the thermal comfort situation of animals with the following scale: up to 74, comfort; from 74 to 78, alert; from 79 to 84, dangerous; and above 84, emergency [32].

The physiological variables studied were the rectal temperature (RT, °C), respiratory rate (RR, movements/minute) and body surface temperature (BST, °C) measured every 14 days, in the morning between 6:00 a.m. and 7:00 a.m. and in the afternoon between 12:00 p.m. and 13:00 p.m. To obtain the RT, a veterinary clinical thermometer was used, with a scale of up to 44 °C, with the reading result expressed in degrees centigrade. The RR was obtained by inspecting and counting the thoracoabdominal movements for 1 min minute. The BST was obtained with the help of an infrared thermometer (Model TD-965—Instrutemp, São Paulo, Brazil) activated at a maximum distance of 1 m from the measurement points on the animal, which were the forehead, left side of the thorax and left flank, having obtained the average of these values. Data for physiological variables were expressed as means and standard deviations.

**Figure 2:**
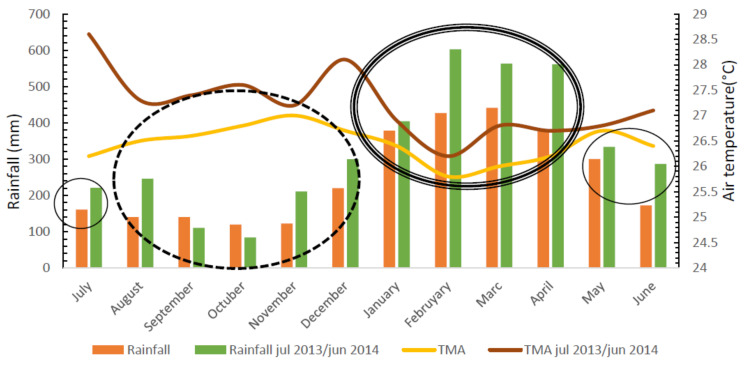
Climatological normal data compared to those observed during the experimental period in the municipality of Belém, Pará. Note: TMA = Average air temperature; TMA Jul 2013/Jul 2014 = average annual temperature.

The research considered the transition period between the periods (more and less rainy) of the year, identified when analyzing the climatic data, mainly in the afternoon, from May to July, where there was a significant decrease in the relative humidity of the air, as well as a significant increase in the air temperature. Thus, the data were organized into three periods of the year: rainier (January to April), transition (May to July) and less rainy (August to December) (Figure 2). The objective of this division was to detect whether the meteorological conditions of these periods influenced the physiological responses of buffaloes.

### 2.5. Statistical Analysis

A completely randomized experimental design was used, in a factorial arrangement of four treatments (0%, 0.5%, 0.25% and 1% of palm kernel cake), three periods of the year (rainier, transition and less rainy) and two shifts (morning and afternoon). Analyses of variance were performed using the Statistical Analysis System software (Version 6.08) [33] to verify the effect of the treatment on the co-product levels, day shift (morning and afternoon), and period of the year (rainier, transition and less rainy), and their interactions with the physiological parameters mentioned above. Means were compared using Tukey’s test, at 5% probability. Pearson’s linear correlations were performed to verify the magnitude and direction of the proportionality of the physiological variables and observe the independence of the variances of the pairs of observations used.

## 3. Results

The average rainfall during the experimental period was 3921.9 mm, the rainiest period being from January to May (monthly rainfall greater than 330 mm), and the least rainy period corresponding to the period from August to December.

The average annual temperature was 26.4 °C, with maximum monthly averages between 30.9 and 32.8 °C, relative air humidity around 84% and annual insolation of 2586 h. The average temperature observed in the less rainy period was equal to 26.7 °C, being higher than that observed in the wettest period (26 °C).

There was a difference in the GTHI between the shifts, with the afternoon averages being higher throughout the year and especially in the less rainy period, where the GTHI reached 92.06 ± 2.74, statistically different from the rainiest period (88.66 ± 3.78) and transitional (87.65 ± 3.76) (*p* < 0.05) (Table 2).

The diets did not influence the rectal temperature (*p* > 0.05), but there was an influence of the environment on this variable (*p* < 0.05). In all periods of the year, the mean RT values in the afternoon shift were higher (rainiest period: 39.23 ± 0.86 °C, transition: 39.00 ± 0.93 °C and less rainy period: 39.15 ± 0.66 °C), compared to those in the morning (mean 38.67 °C) (Table 3). In both shifts, there was no difference in RT between periods of the year (*p* > 0.05).

Supplementation also did not influence respiratory rate values (*p* > 0.05). However, in all periods of the year, there was a difference between shifts (*p* < 0.05). The RR of the afternoon shift (wettest period: 49.40 ± 28.75 mov./min., transition: 40.16 ± 22.15 mov./min. and less rainy: 35.66 ± 17.08 mov./min.) was always higher (*p* < 0.05). The effect of the periods of the year on the RR values of the afternoon shift, where the highest RR means occurred in the wettest period of the year (*p* < 0.05).

The body surface temperature values were not influenced, which were also not influenced by the period of the year (*p* > 0.05). The BST was influenced by the shifts, with the highest values also observed in the afternoon (rainiest period: 34.46 ± 2.07 °C, transition: 34.30 ± 1.72 °C and least rainy: 34.82 ± 1.23 °C) (*p* < 0.05).

The RT showed a positive correlation with air temperature (0.628) and GTHI (0.597), and a negative correlation with relative air humidity (−0.569) (Table 4). RR also showed a positive correlation with air temperature (0.748) and GTHI (0.689), and a negative correlation with relative air humidity (−0.637). In BST, the correlation was positive with air temperature (0.939) and GTHI (0.933), and negative with relative humidity (−0.873).

## 4. Discussion

Precipitation data during the experimental period remained within the local average, and are also in line with those found by Pachêco [34]. In this study, palm oil cake did not influence the thermoregulation of buffaloes in the Amazon. The thermal comfort of the animals depends, to a high degree, on the levels of relative humidity in the air in association with the air temperature [6,17], and the GTHI represents this association well.

The GTHI differences between the shifts (all periods of the year), with higher averages in the afternoon shift, indicate an emergency situation for the animals, suggesting thermal stress, especially in the less rainy period of the year, where the GTHI had the highest average. Similar GTHI values were found by Silva et al. [35], with averages in the afternoon shift of 83.9 ± 2.2, 87.6 ± 3.1 and 88.8 ± 2.6, in the rainiest, transition and less rainy periods of the year, respectively, when evaluating the thermal comfort of buffaloes in a silvopastoral system, also in the eastern Amazon. The results were also close to those recorded by Santos et al. [36] in the Brazilian semi-arid region (85.5—afternoon shift). Apart from certain GTHI or THI, thermoregulatory mechanisms to deal with thermal stress are not sufficient to maintain thermal stability [37].

The absence of significance in the thermoregulatory responses of the animals probably occurred because the animals had presented moderate thermal stress [38]. However, the animals suffered opposite effects, where in the afternoon, with high values of GTHI and air temperature, the efficiency of heat loss by conduction, convection and radiation decreased, due to the increase in the temperature gradient between the animal’s skin and the environment. In this situation, the animal can, to a certain extent, maintain body temperature through peripheral vasodilation, increasing peripheral blood flow and surface temperature [39]. However, if the ambient temperature continues to rise, the animal starts to depend on the loss of heat by evaporation, through respiration (increase in RR), to maintain homeothermia [40].

The elevation of rectal temperature in a hot environment indicates that the heat release mechanisms have become insufficient to maintain homeothermia [41,42]. This result can be attributed to the higher incidence of solar radiation in the afternoon. Cattle and buffaloes, in similar climatic conditions, presented similar results [43], where the RT of buffaloes increased from 38.0 °C to 39.3 °C, in the morning and afternoon, respectively, reaffirming that this variable physiology is influenced by the rise in ambient temperature. Higher rectal temperatures in the afternoon were also observed by Magalhães et al. [43] and Silva et al. [44], who found the RT (39.26 and 39.11 °C) of cattle and buffaloes in the afternoon higher than those in the morning (38.05 and 38.11 °C), respectively. The variation in rectal temperature in crossbred buffaloes was observed by Shenhe et al. [45] during the seasons of the year, and they also found the influence of environmental temperature: (39.24 °C) in summer; (38.34 °C) in spring and (38.13 °C) in winter. This increase can be attributed to the inability to dissipate excess body heat generated during the thermal load of the warmer months [46,47].

Even in the morning (milder temperatures), the values are above the normal variation range for buffaloes, from 37.4 to 37.9 °C [47]. The effects of ambient temperature on the rectal temperature of buffaloes are an indication of thermal stress in animals [48,49]. An increase of close to 1 °C in rectal temperature is enough to reduce productive performance in most domestic animal species [1]. In both shifts, there was no difference in RT (*p* > 0.05) between the periods of the year, demonstrating that in all periods of the year in the Amazon region, the animals are susceptible to thermal stress in the afternoon shift.

In all periods of the year, the RR of the afternoon shift was higher. This was probably due to the fact that it was the hottest period of the day, and due to the onset of respiratory evaporative thermolysis mechanisms in order to maintain homeothermia. In evaluating the mean RR data, in the morning shift, the observed values are within the normal variation range for buffaloes, from 18 to 30 (movements/minute) [46]. In the same region (eastern Amazon), Silva et al. [44] observed higher values in buffalo heifers in the morning shift (28.3 ± 1.1 to 29.8 ± 1.1—movements/minute), but still within the normal variation range for buffaloes.

On the other hand, the values observed in the afternoon (all periods of the year), surpassed those indicated for situations of thermoneutrality of the buffalo species, which was already expected, since the combination of climatic elements caused a greater degree of discomfort to the animals, because they raised the respiratory rate in order to maintain the body temperature. In situations of thermal stress, this physiological variable presents high values, even before there is an increase in rectal temperature values [49,50]. In an attempt to maintain body temperature, the buffaloes increased their respiratory rate from 22 to 48 movements per min, when the temperature ranged from 28.3 to 34.7 °C [51].

In the afternoon shift, the highest RR values occurred in the wettest period of the year. This result may have occurred due to the difficulty these animals have in dissipating heat by evaporation, as the RH is quite high during this period. In buffaloes, heat loss through exhaled air is more important than perspiration, as they have low efficiency in heat loss through the skin [52,53].

The information obtained from BST has been widely used, as in addition to helping in the diagnosis of pathologies, it can indicate the physiological state and well-being of the animals [54]. The effect of the day shift on BST occurred as the highest GTHI and AT values were found in the afternoon. Because of this, there was an increase in blood flow from the central core to the body periphery, in an attempt to eliminate heat, which contributed to the elevation of BST [35,55]. BST is directly related to the environmental conditions of RH, air temperature, incidence of wind, and physiological conditions, such as vascularization and evaporation by sweat [50,56]. At mild air temperatures, this variable contributes to maintaining body temperature through heat exchange with the environment [57].

The correlation data suggest that increasing air temperature increases RT, RR and BST, that the increase in RH implies a reduction in RT, RR and BST, and the increase in GTHI reflects the elevation of RT, RR and BST data. The positive correlation between air temperature and GTHI, and the negative one with relative humidity, indicates that the animals reacted to increases in air temperature and GTHI with an increase in RT. The conditions of climate, environment and diet influence the metabolism and physiology of the animals, which may be reflected in the increase in rectal temperature as a response to the situation [58].

The correlation verified for RR reinforces the idea of regulatory changes depending on the environment. The clearest physiological effects of heat stress in animals include pronounced changes in their heart rate and respiratory rate [59].

The dynamics for BST demonstrate that under conditions with high ambient temperatures and high GTHI, the animal tries to dissipate excess heat and increases blood flow from the central nucleus to the body surface and, consequently, increases the rate of heat flow, which results in high reductions in temperature [60]. When AT increases, UR decreases; thus, a negative correlation of UR with BST occurs. Similar results were obtained by Silva et al. [35], demonstrating that this variable is an excellent indicator of heat stress in buffaloes.

**Figure 3:**
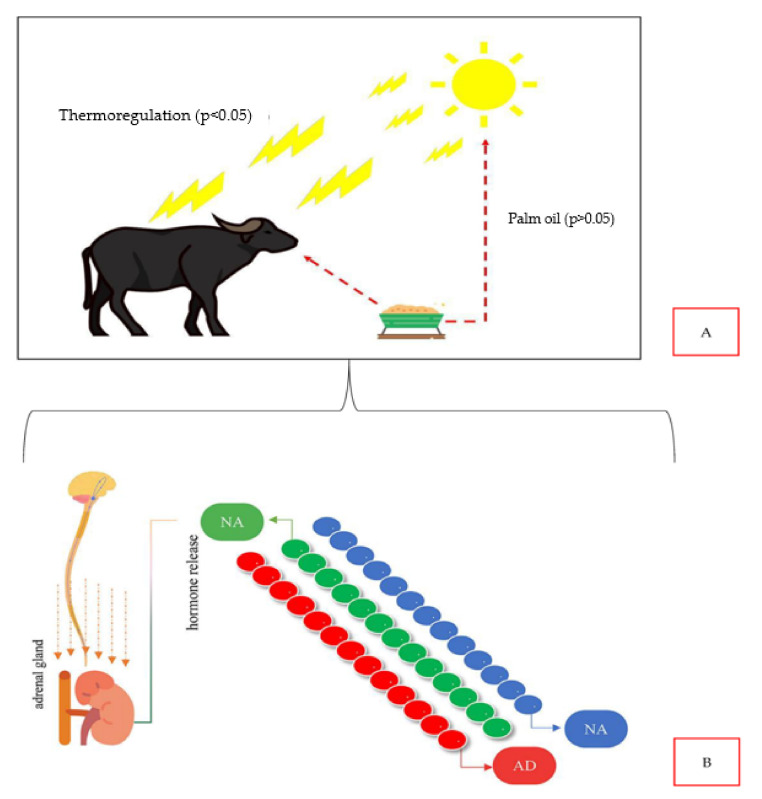
(**A**) Experiment result infographic (the authors). (**B**) Graphic representation of the stress stimulus in buffaloes that triggers the release of catecholamines by the sympathetic nervous system, namely adrenaline (AD) and noradrenaline (NAD), as well as cortisol (CT) by the adrenal gland Adapted from [6].

Furthermore, the fact that supplementation with palm kernel cake did not influence the thermoregulation of buffaloes in the eastern Amazon is an extremely important result (Figure 3). As mentioned, the regions influenced by the Amazonian climate, generally, have six months of the year with high rainfall intensity [17,61,62,63,64] as well as the availability of food in the pasture. However, the other six months are of low rainfall (dry period), where forage availability and quality is reduced. During this period, there is a need for supplementation, so that the animals do not decrease their performance or even lose weight. With the information obtained in this work, we can indicate the co-product (palm kernel cake), abundant in the region, as a food strategy in dry periods. These are data that could directly influence the buffalo production chain in the Amazon region.

## 5. Conclusions

In conclusion, the study reveals that environmental factors, such as the time of day (morning vs. afternoon), air temperature, Global Temperature and Humidity Index (GTHI), and relative humidity, exert a more pronounced influence on physiological parameters including the rectal temperature (RT), respiratory rate (RR), and body surface temperature (BST), compared to dietary supplementation or the specific period of the year. Notably, afternoon shifts consistently exhibit higher RT and BST values, indicating the critical role of time and environmental conditions in shaping these responses. While dietary supplementation with palm oil cake did not significantly impact thermoregulatory responses in buffalo within the eastern Amazon’s climatic conditions, it presents a potential avenue to enhance productivity. Nevertheless, the study underscores the substantial impact of weather conditions on animal well-being. Further research is recommended to optimize the utilization of these co-products in supplementation in buffalo in the Amazon region.

## Figures and Tables

**Table 1:** Chemical composition of ingredients and diets.

Diet	Components (% DM)					
	DM	Ash	EE	CP	NDF	ADF
Palm kernel cake	90.47	4.61	11.64	11.12	69.87	48.23
Wheat bran	88.32	5.88	3.48	15.49	44.19	14.27
*Brachiaria brizantha*	37.4	6.76	2.54	8.19	68.14	40.55
0%	88.32	5.88	3.48	11.69	44.19	14.27
0.25%	89.66	5.09	8.58	12.13	60.24	35.5
0.50%	89.97	4.9	9.75	12.76	63.94	40.39
1%	90.17	4.78	10.57	15.49	66.51	43.78

Dry matter (DM); ether extract (EE); crude protein (CP); neutral detergent fiber (NDF); acid detergent fiber (ADF).

**Table 2:** Averages and standard deviation of the Globe Temperature and Humidity Index in the experimental area, in the morning and afternoon shifts, and in the three periods of the year.

Period	Shift	
	Morning	Afternoon
Rainier	74.11 ± 1.09 ^Aa^	88.66 ± 3.78 ^bA^
Transition	74.79 ± 1.27 ^aA^	87.65 ± 3.76 ^bA^
Less rainy	74.46 ± 1.37 ^aA^	92.06 ± 2.74 ^bB^

^A,B^ Period averages, within each shift, followed by different capital letters, in the same column of each climate variable and index are different (*p* < 0.05); ^a,b^ Means of shifts, within each period, followed by different lowercase letters, in the same line are different (*p* < 0.05).

**Table 3:** Means and standard deviations of physiological variables in the morning and afternoon, in the three periods of the year.

Physiological Variable	Period	Shift	
		Morning	Afternoon
Rectal temperature (°C)	Rainier	38.64 ± 0.36 ^aA^	39.23 ± 0.86 ^bA^
	Transition	38.62 ± 0.22 ^aA^	39.00 ± 0.93 ^bA^
	Less rainy	38.77 ± 0.70 ^aA^	39.15 ± 0.66 ^bA^
Respiratory rate (mov./min.)	Rainier	19.95 ± 5.94 ^aA^	49.40 ± 28.75 ^bA^
	Transition	19.05 ± 5.39 ^aA^	40.16 ± 22.15 ^bAB^
	Less rainy	19.94 ± 7.04 ^aA^	35.66 ± 17.08 ^aB^
Body surface temperature (°C)	Rainier	30.46 ± 1.34 ^aA^	34.46 ± 2.07 ^bA^
	Transition	29.55 ± 1.33 ^aA^	34.30 ± 1.72 ^bA^
	Less rainy	29.84 ± 1.41 ^aA^	34.82 ± 1.23 ^bA^

^A,B^ Period averages, within each shift, followed by different capital letters, in the same column of each physiological variable, are different (*p* < 0.05). ^a,b^ Shift averages, within each period, followed by different lowercase letters, on the same line are different (*p* < 0.05).

**Table 4:** Correlation between climatic variables, thermal comfort and physiological indices of crossbreeds (Murrah x Mediterranean) buffaloes.

Index	RT (°C)	RR (mov./min.)	BST (°C)
Air temperature	0.628 **	0.748 **	0.939 **
Relative humidity	−0.569 **	−0.637 **	−0.873 **
Globe Temperature and Humidity Index	0.597 **	0.689 **	0.933 **

RT—Rectal temperature; BST—Body surface temperature; RR—Respiratory rate (movements per minute). **—significance at 1%.

## Data Availability

The data presented in this study are available upon reasonable request from the corresponding author.

## References

[1] McDowell R.E., Hooven N.W., Camoens J.K. (1976). Effect of climate on performance of Holsteins in first lactation. J. Dairy Sci..

[2] Abduch N.G., Pires B.V., Souza L.L., Vicentini R.R., Zadra L.E.F., Fragomeni B.O., Silva R.M.O., Baldi F., Paz C.C.P., Stafuzza N.B. (2022). Effect of Thermal Stress on Thermoregulation, Hematological and Hormonal Characteristics of Caracu Beef Cattle. Animals.

[3] Lima A.R.C., Silveira R.M.F., Castro M.S.M., De Vecchi L.B., da Fernandes M.H.M.R., Resende K.T. (2022). Relationship between thermal environment, thermoregulatory responses and energy metabolism in goats: A comprehensive review. J. Therm. Biol..

[4] Dos Santos M.M., Souza-Junior J.B.F., Dantas M.R.T., de Macedo Costa L.L. (2021). An updated review on cattle thermoregulation: Physiological responses, biophysical mechanisms, and heat stress alleviation pathways. Environ. Sci. Pollut. Res..

[5] Marai I.F.M., Haeeb A.A.M. (2010). Buffalo’s biological functions as affected by heat stress—A review. Livest. Sci..

[6] Silva W.C.D., Silva J.A.R.D., Camargo-Júnior R.N.C., Silva É.B.R.D., Santos M.R.P.D., Viana R.B., Silva C.M.G.D., Lourenço-Júnior J.D.B. (2023). Animal welfare and effects of per-female stress on male and cattle reproduction—A review. Front. Vet. Sci..

[7] Silva W.C.D., Silva J.A.R.D., Silva É.B.R.D., Barbosa A.V.C., Sousa C.E.L., Carvalho K.C.D., Santos M.R.P.D., Neves K.A.L., Martorano L.G., Camargo Júnior R.N.C. (2023). Characterization of Thermal Patterns Using Infrared Thermography and Thermolytic Responses of Cattle Reared in Three Different Systems during the Transition Period in the Eastern Amazon, Brazil. Animals.

[8] Colombo E.A., Cooke R.F., Millican A.A., Schubach K.M., Scatolin G.N., Rett B., Brandão A.P. (2019). Supplementing an immunomodulatory feed ingredient to improve thermoregulation and performance of finishing beef cattle under heat stress conditions. J. Anim. Sci..

[9] Somagond Y.M., Singh S.V., Deshpande A., Sheoran P., Chahal V.P. (2021). Infrared thermography to assess thermoregulatory reactions of buffaloes supplemented with antioxidant and dense energy source in summer season. J. Agrometeorol..

[10] Silveira R.M.F., Silva B.E.B.E., de Vasconcelos A.M., Façanha D.A.E., Martins T.P., Rogério M.C.P., Ferreira J. (2021). Does organic selenium supplement affect the thermoregulatory responses of dairy goats?. Biol. Rhythm Res..

[11] Henry M.L., Kemp S., Clarke I.J., Dunshea F.R., Leury B.J. (2019). Perennial ryegrass alkaloids increase respiration rate and decrease plasma prolactin in merino sheep under both thermoneutral and mild heat conditions. Toxins.

[12] Prathap P., Chauhan S.S., Leury B.J., Cottrell J.J., Joy A., Zhang M., Dunshea F.R. (2022). Reducing the fermentability of wheat with a starch binding agent reduces some of the negative effects of heat stress in sheep. Animals.

[13] Ding J., He S., Xiong Y., Liu D., Dai S., Hu H. (2020). Effects of dietary supplementation of fumaric acid on growth performance, blood hematological and biochemical profile of broiler chickens exposed to chronic heat stress. Braz. J. Poult. Sci..

[14] Serviento A.M., Labussière E., Castex M., Renaudeau D. (2020). Effect of heat stress and feeding management on growth performance and physiological responses of finishing pigs. J. Anim. Sci..

[15] Adepará (2023). Agencia de Defesa Agropecuário do Estado do Pará. Estado do Pará Detém o Segundo Maior Rebanho Bovino do Brasil e o Maior de Búfalos. http://www.adepara.pa.gov.br/artigos.

[16] Forti M.C., Melfi A.J., Astolfo R., Fostier A.H. (2000). Rainfall chemistry composition in two ecosystems in the northeastern Brazilian Amazon (Amapá State). J. Geophys. Res. Atmos..

[17] Algra M., de Keijzer L., Arndt S.S., van Eerdenburg F.J., Goerlich V.C. (2023). Evaluation of the Thermal Response of the Horns in Dairy Cattle. Animals.

[18] Rueda B.L., McRoberts K.C., Blake R.W., Nicholson C.F., Valentim J.F., Fernandes E.C.M. (2020). Nutrient status of cattle grazing systems in the western brazilian amazon. Cogent Food Agric..

[19] Chong C.H., Zulkifli I., Blair R. (2008). Effects of dietary inclusion of palm kernel cake and palm oil, and enzyme supplementation on performance of laying hens. Asian-Australas. J. Anim. Sci..

[20] Soares C., Rossa F., da Silva F.F., da Silva A.P., Santos L.V., de Lima Júnior D.M., Silva R.R. (2022). Effect of palm kernel cake inclusion in the supplement of pasture-finished heifers on performance, carcass traits, and meat quality. N. Z. J. Agric. Res..

[21] Ferreira F.G., Leite L.C., Alba H.D., Pina D.D.S., Santos S.A., Tosto M.S., Rodrigues C.S., de Lima Júnior D.M., de Oliveira J.S., de Freitas Júnior J.E. (2022). Palm kernel cake in diets for lactating goats: Intake, digestibility, feeding behavior, milk production, and nitrogen metabolism. Animals.

[22] Alford A.R., Hegarty R.S., Parnell P.F., Cacho O.J., Herd R.M., Griffith G.R. (2006). The impact of breeding to reduce residual feed intake on enteric methane emissions from the Australian beef industry. Aust. J. Exp. Agric..

[23] Freitas T.B., Felix T.L., Pedreira M.S., Silva R.R., Silva F.F., Silva H.G.O., Moreira B.S. (2017). Effects of increasing palm kernel cake inclusion in supplements fed to grazing lambs on growth performance, carcass characteristics, and fatty acid profile. Anim. Feed Sci. Technol..

[24] Alemu A.W., Vyas D., Manafiazar G., Basarab J.A., Beauchemin K.A. (2017). Enteric methane emissions from low–and high–residual feed intake beef heifers measured using GreenFeed and respiration chamber techniques. J. Anim. Sci..

[25] Mota-Rojas D., Titto C.G., de Mira Geraldo A., Martínez-Burnes J., Gómez J., Hernández-Ávalos I., Casas A., Domínguez A., José N., Bertoni A. (2021). Efficacy and function of feathers, hair, and glabrous skin in the thermoregulation strategies of domestic animals. Animals.

[26] Bertoni A., Napolitano F., Mota-Rojas D., Sabia E., Álvarez-Macías A., Mora-Medina P., Morales-Canela A., Berdugo-Gutiérrez J., Guerrero-Legarreta I. (2020). Similarities and differences between river buffaloes and cattle: Health, physiological, behavioral and productivity aspects. J. Buffalo Sci..

[27] Martorano L.G., Nechet D., Pereira L.C. (1993). Tipologia climática do Estado do Pará: Adaptação do método de Köppen. Bol. Geogr. Teorética.

[28] AOAC (2005). Official Methods of Analysis of the Association Analytical Chemists.

[29] Van Soest P.J., Robertson J.B., Lewis B.A. (1991). Methods for dietary fiber, neutral detergent fiber, and nonstarch polysaccharides in relation to animal nutrition. J. Dairy Sci..

[30] Buffington D.E., Collazo Arocho A., Canton G.H., Pitt D. (1981). Black globe humidity index (BGHI) as a comfort equation for dairy cows. Trans. ASAE.

[31] Marcheto F.G., Nääs I.D.A., Salgado D.D.A., Souza S.R.L.D. (2002). Efeito das temperaturas de bulbo seco e de globo negro e do índice de temperatura e umidade, em vacas em produção alojadas em sistema de free-stall. Braz. J. Vet. Res. Anim. Sci..

[32] Souza C.D.F., Tinôco I.D.F., Baêta F.D.C., Ferreira W.P.M., Silva R.D. (2002). Avaliação de materiais alternativos para confecção de termômetro de globo. Ciência Agrotecnologia.

[33] Statistical Analysis System—SAS (2003). SAS User’s Guide.

[34] Pachêco N.A., Santiago A.V., Bastos T.X., Cordeiro A.H.F. (2009). Boletim Agrometerorológico de 2009 para Belém, P.A. Documentos, 371.

[35] Silva J.A.R.D., Araújo A.A.D., Lourenço Júnior J.D.B., Santos N.D.F.A.D., Garcia A.R., Nahúm B.D.S. (2011). Conforto térmico de búfalas em sistema silvipastoril na Amazônia Oriental. Pesqui. Agropecuária Bras..

[36] Santos F.C.B.D., Souza B.B.D., Peña Alfaro C.E., Cézar M.F., Pimenta Filho E.C., Acosta A.A.A., Santos J.R.S.D. (2005). Adaptabilidade de caprinos exóticos e naturalizados ao clima semi-árido do Nordeste brasileiro. Ciência Agrotecnologia.

[37] Silanikove N. (2000). Effects of heat stress on the welfare of extensively managed domestic ruminants. Livest. Prod. Sci..

[38] Dash S., Chakravarty A.K., Singh A., Upadhyay A., Singh M., Yousuf S. (2016). Effect of heat stress on reproductive performances of dairy cattle and buffaloes: A review. Vet. World.

[39] Lendez P.A., Cuesta L.M., Farias M.V.N., Vater A.A., Ghezzi M.D., Mota-Rojas D., Dolcini G.L., Ceriani M.C. (2021). Alterations in TNF-α and its receptors expression in cows undergoing heat stress. Vet. Immunol. Immunopathol..

[40] Mishra S.R. (2021). Thermoregulatory responses in riverine buffaloes against heat stress: An updated review. J. Therm. Biol..

[41] Robinson N.E., Cunningham J.G. (2004). Homeostase—Termorregulação. Tratado de Fisiologia Veterinária.

[42] Idris M., Uddin J., Sullivan M., McNeill D.M., Phillips C.J. (2021). Non-invasive physiological indicators of heat stress in cattle. Animals.

[43] Magalhães J.A., Takigawa R.M., Townsend C.R., Costa N.D.L., Pereira R.D.A. (2000). Tolerância de bovídeos à Temperatura e Umidade do Trópico Úmido. Rev. Científica Produção Anim..

[44] Silva J.A.R., Araújo A.A., Lourenço Júnior J.D.B., Viana R.B., Santos N.D.F.A., Garcia A.R. (2011). Perfil hematológico de búfalas da raça Murrah, criadas ao sol e à sombra, em clima tropical da Amazônia Oriental. Acta Amaz..

[45] Shenhe L., Jun L., Zipeng L., Tingxian D., ur Rehman Z., Zichao Z., Liguo Y. (2018). Effect of season and breed on physiological and blood parameters in buffaloes. J. Dairy Res..

[46] Shafie M.M., Yousef M.K. (2000). Physiology responses adaptation of water buffalo. MK Stress Physiology in Livestock, Volume 2, Ungulates.

[47] Mishra S.R. (2021). Behavioural, physiological, neuro-endocrine and molecular responses of cattle against heat stress: An updated review. Trop. Anim. Health Prod..

[48] Chikamune T., Shimizu H. (1983). Comparison of physiological response to climatic conditions in swamp buffaloes and cattle. Indian J. Anim. Sci..

[49] Ferreira F., Pires M.F.A., Martinez M.L., Coelho S.G., Carvalho A.U., Ferreira P.M., Facury Filho E.J., Campos W.E. (2006). Parâmetros fisiológicos de bovinos cruzados submetidos ao estresse calórico. Arq. Bras. Med. Veterinária Zootec..

[50] Berihulay H., Abied A., He X., Jiang L., Ma Y. (2019). Adaptation mechanisms of small ruminants to environmental heat stress. Animals.

[51] Guimarães C.M.C., Falco J.E., Titto E.A.L., Franzolin Neto R., Muniz J.A. (2001). Termorregulação em bubalinos submetidos a duas temperaturas de ar e duas proporções de volumoso:concentrado. Ciência Agrotecnologia.

[52] Mota-Rojas D., Napolitano F., Braghieri A., Guerrero-Legarreta I., Bertoni A., Martínez-Burnes J., Cruz-Monterrosa R., Gómez J., Ramírez-Bribiesca E., Barrios-García H. (2020). Thermal biology in river buffalo in the humid tropics: Neurophysiological and behavioral responses assessed by infrared thermography. J. Anim. Behav. Biometeorol..

[53] Fu M., Weng W., Chen W., Luo N. (2016). Review on modeling heat transfer and thermoregulatory responses in human body. J. Therm. Biol..

[54] Mota-Rojas D., Pereira A.M., Wang D., Martínez-Burnes J., Ghezzi M., Hernández-Avalos I., Lendez P., Mora-Medina P., Casas A., Olmos-Hernández A. (2021). Clinical applications and factors involved in validating thermal windows used in infrared thermography in cattle and river buffalo to assess health and productivity. Animals.

[55] Li M., Liang X., Tang Z., Hassan F.U., Li L., Guo Y., Peng K., Liang X., Yang C. (2021). Thermal comfort index for lactating water buffaloes under hot and humid climate. Animals.

[56] Mota-Rojas D., Titto C.G., Orihuela A., Martínez-Burnes J., Gómez-Prado J., Torres-Bernal F., Flores-Padilla K., Carvajal-de la Fuente V., Wang D. (2021). Physiological and behavioral mechanisms of thermoregulation in mammals. Animals.

[57] Brcko C.C., Silva J.A.R.D., Martorano L.G., Vilela R.A., Nahúm B.D.S., Silva A.G.M., Barbosa A.V.C., Bezerra A.S., Lourenço Júnior J.D.B. (2020). Infrared thermography to assess thermoregulatory reactions of female buffaloes in a humid tropical environment. Front. Vet. Sci..

[58] Petrocchi Jasinski F., Evangelista C., Basiricò L., Bernabucci U. (2023). Responses of Dairy Buffalo to Heat Stress Conditions and Mitigation Strategies: A Review. Animals.

[59] Li M., Hassan F.U., Guo Y., Tang Z., Liang X., Xie F., Peng L., Yang C. (2020). Seasonal dynamics of physiological, oxidative and metabolic responses in non-lactating Nili-Ravi buffaloes under hot and humid climate. Front. Vet. Sci..

[60] Vittorazzi P.C., Takiya C.S., Nunes A.T., Chesini R.G., Bugoni M., Silva G.G., Silva T.B., Dias M.S., Grigoletto N.T., Rennó F.P. (2022). Feeding encapsulated pepper to dairy cows during the hot season improves performance without affecting core and skin temperature. J. Dairy Sci..

[61] Silva J.A.R.D., Pantoja M.H.D.A., Silva W.C.D., Almeida J.C.F.D., Noronha R.D.P.P., Barbosa A.V.C., Lourenço Júnior J.D.B. (2022). Thermoregulatory reactions of female buffaloes raised in the sun and in the shade, in the climatic conditions of the rainy season of the Island of Marajó, Pará, Brazil. Front. Vet. Sci..

[62] Silva W.C.D., Silva É.B.R.D., Santos M.R.P.D., Camargo Junior R.N.C., Barbosa A.V.C., Silva J.A.R.D., Vinhote J.A., Sousa E.D.V.D., Lourenço Júnior J.D.B. (2022). Behavior and thermal comfort of light and dark coat dairy cows in the Eastern Amazon. Front. Vet. Sci..

[63] Da Silva W.C., Printes O.V.N., Lima D.O., da Silva É.B.R., Dos Santos M.R.P., Júnior R.N.C.C., Barbosa A.V.C., da Silva J.A.R., e Silva A.G.M., Silva L.K.X. (2023). Evaluation of the temperature and humidity index to support the implementation of a rearing system for ruminants in the Western Amazon. Front. Vet. Sci..

[64] Da Silva W.C., Rodrigues J.A., de Alvarenga A.B.B., Barbosa A.V.C., da Silva É.B.R., dos Santos M.R.P., de Brito Lourenço-Júnior J., Júnior R.N.C.C., e Silva A.G.M. (2023). New proposal for the use of the focal animal technique in buffaloes in the Eastern Amazon. Front. Vet. Sci..

